# A tangled threesome: understanding arbovirus infection in *Aedes* spp. and the effect of the mosquito microbiota

**DOI:** 10.3389/fmicb.2023.1287519

**Published:** 2024-01-03

**Authors:** Juan S. Mantilla-Granados, Jaime E. Castellanos, Myriam Lucía Velandia-Romero

**Affiliations:** ^1^Saneamiento Ecológico, Salud y Medio Ambiente, Universidad El Bosque, Vicerrectoría de Investigaciones, Bogotá, Colombia; ^2^Grupo de Virología, Universidad El Bosque, Vicerrectoría de Investigaciones, Bogotá, Colombia

**Keywords:** *Aedes aegypti*, *Aedes albopictus*, vectorial competence, extracellular vesicles, RNA interference, insect specific virus, mosquito-borne disease, Wolbachia

## Abstract

Arboviral infections transmitted by *Aedes* spp. mosquitoes are a major threat to human health, particularly in tropical regions but are expanding to temperate regions. The ability of *Aedes aegypti* and *Aedes albopictus* to transmit multiple arboviruses involves a complex relationship between mosquitoes and the virus, with recent discoveries shedding light on it. Furthermore, this relationship is not solely between mosquitoes and arboviruses, but also involves the mosquito microbiome. Here, we aimed to construct a comprehensive review of the latest information about the arbovirus infection process in *A. aegypti* and *A. albopictus*, the source of mosquito microbiota, and its interaction with the arbovirus infection process, in terms of its implications for vectorial competence. First, we summarized studies showing a new mechanism for arbovirus infection at the cellular level, recently described innate immunological pathways, and the mechanism of adaptive response in mosquitoes. Second, we addressed the general sources of the *Aedes* mosquito microbiota (bacteria, fungi, and viruses) during their life cycle, and the geographical reports of the most common microbiota in adults mosquitoes. How the microbiota interacts directly or indirectly with arbovirus transmission, thereby modifying vectorial competence. We highlight the complexity of this tripartite relationship, influenced by intrinsic and extrinsic conditions at different geographical scales, with many gaps to fill and promising directions for developing strategies to control arbovirus transmission and to gain a better understanding of vectorial competence. The interactions between mosquitoes, arboviruses and their associated microbiota are yet to be investigated in depth.

## 1 Introduction

Only ~1% of *Aedes* spp. are involved in transmitting pathogens affecting human health. *Aedes aegypti* and *Aedes albopictus* are the main vectors of arboviruses, such as dengue (DENV), Zika (ZIKV), yellow fever (YFV), and chikungunya (CHIKV) (Wilkerson et al., 2015; Houé et al., 2019a). Their role as vectors is linked to their ability to allow viral development, become infected, and then transmit it to a susceptible host, known as vectorial competence, and the ability of the virus to overcome the mosquito's immune response (Moncada et al., 2021). The mosquito microbiota (bacteria, fungi, and viruses) can influence the complex relationship between mosquitoes and arboviruses (Vega-Rua et al., 2014; Pang et al., 2016; Caragata et al., 2019; Houé et al., 2019b; Yin et al., 2020). Understanding this relationship is becoming increasingly important, as it is a great source of information for developing effective strategies to control arbovirus transmission. This review identifies the generalities of arbovirus infection in mosquito's cells and *Aedes* immune responses to arboviral infections, focusing on the most recent discoveries. We then discuss the presence and sources of their microbiota and how this affects their vectorial competence; we also identify the knowledge gaps and suggest perspectives for future research.

## 2 Mosquitoes and arbovirus

### 2.1 Arbovirus infection in mosquitoes and insect cells

Arbovirus acquisition in mosquitoes begins with the ingestion of an infected blood meal. Once in the midgut, the viral particles are exposed to trypsin produced during blood digestion (Noriega and Wells, 1999), which destroys the viral envelope. Despite that the peritrophic matrix (PM) does not act as a barrier for arbovirus infections, a peroxidase that mediates PM formation, enhance arbovirus infections through oxidative stress regulation (Talyuli et al., 2023), to infect the midgut epithelial cells (the first target of infection), the virus must first pass through the cellular mucin layer. Midgut cells and their immunological responses contribute to the establishment of a midgut infection barrier (MIB) (Moncada et al., 2021).

Once the virus escapes the MIB (dissemination capacity), it enters the hemocoel (open circulatory system in the mosquito) and can infect circulatory cells, including granulocytes (the main phagocytic cells), enocytes (involved in melanization), and prohemocytes (stem cells with phagocytic capacity) (Castillo et al., 2006). Prohemocytes are the main targets of DENV infection (Cheng et al., 2022), suggesting their role as viral amplifiers. In addition, the expression of specific lectins by hemocytes favors the infection of other tissue cells (Cardoso-Jaime et al., 2022), leading to their spread to the adipose tissue, nervous system where the viral infection is controlled by the neural factor Hikaru genki (AaHig) to avoid deleterious effects on the mosquito (Xiao et al., 2015), and ovarioles (involved in vertical transmission). Infection of salivary gland epithelial cells and escape to saliva by passing through the salivary gland infection barrier (SGIB) allows for viral inoculation during the next bite (Moncada et al., 2021). Recently, it has been proposed that mosquito bites may inject not only viral particles but also extracellular vesicles containing viral RNAs or proteins that could infect or modulate the infection of vertebrate cells, changing our understanding of the infection process.

### 2.2 Viral infection in mosquito cells

**Figure 1:**
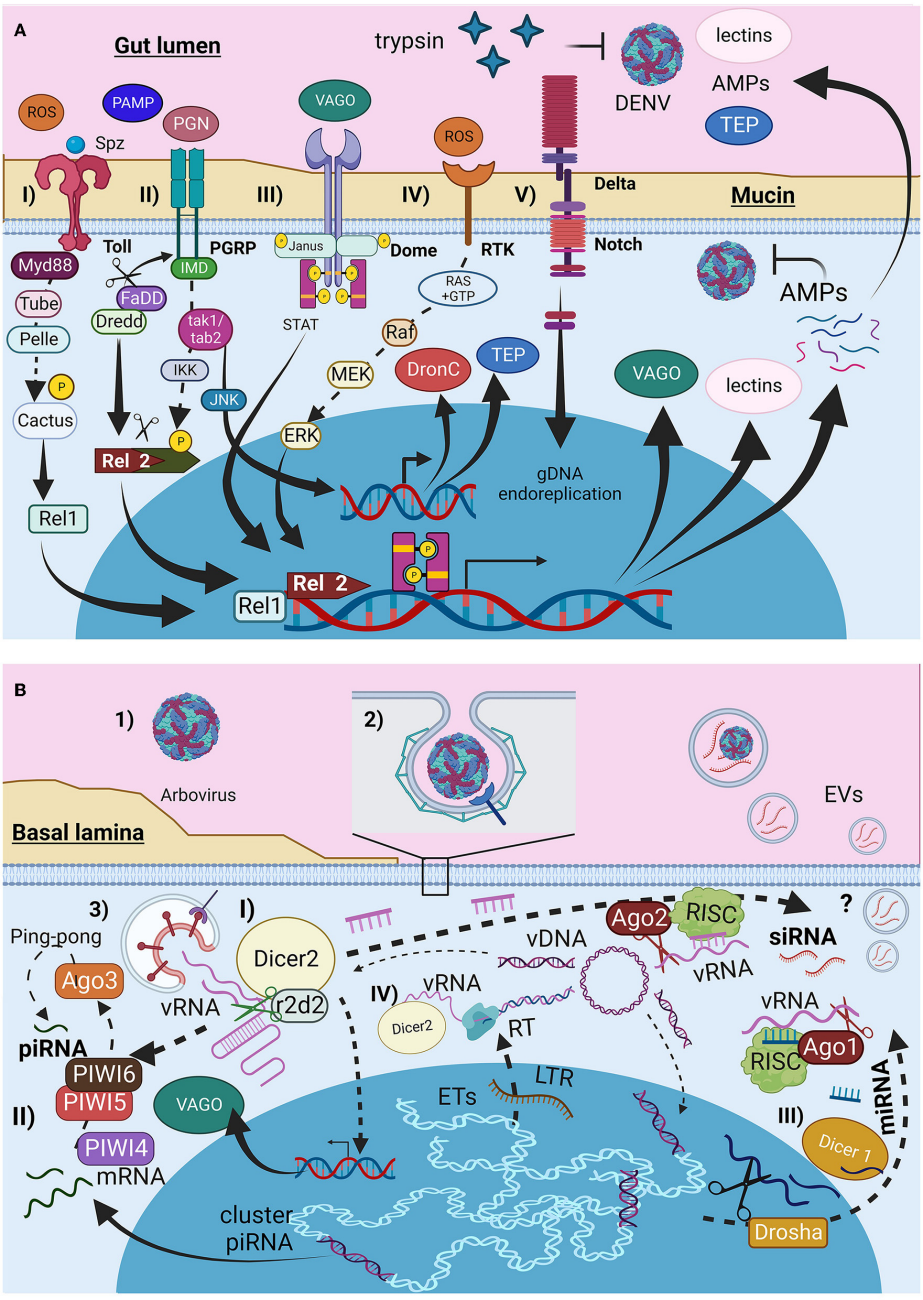
Infection barriers and immunological mosquito cellular response to arbovirus infection. **(A)** Immunological signal pathways (I) Toll receptors, (II) IMD, (III) Dome (JAK-STAT), (IV) tyrosine-kinase receptors (RTK), and (V) Notch pathway. **(B)** Viral entry and RNAi responses produced by mosquitoes cells. (I) small interference RNAs siRNA, (II) Piwi RNAs piRNA, and (III) microRNAs miRNA. Figure produced in BioRender.com.

**Figure 2:**
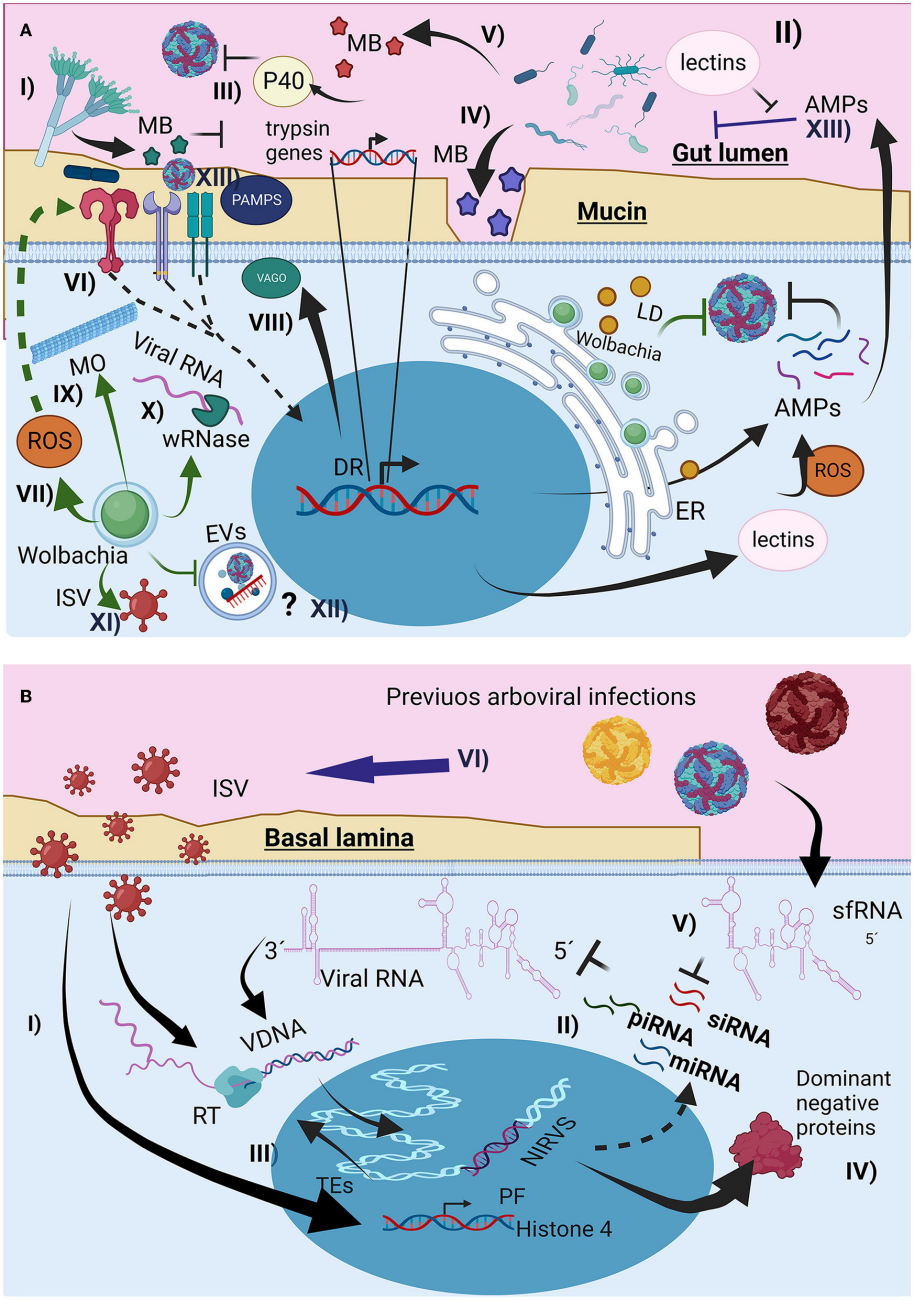
The interaction of the microbiota with the mosquito immunological response affects arboviral infection. **(A)** Direct and indirect mechanism of the microorganism could interfere with arbovirus infection, (I) through metabolites (MB) lumen fungus could affect trypsin production, favoring arbovirus survival, (II) change in lectin production, affecting AMPs recognition, and arbovirus entrance (III) production of proteins like p40 facilitating arbovirus recognition by host cells, (IV) MB production that destroy arbovirus envelope, (V) MB that degrade mucin layer, (VI) Immune priming through canonical immunological pathways, increasing AMPs and ROS production, (VII) Wolbachia presence increase oxidative stress, activating Toll pathway, (VIII) Microtubules reorganization induced by Wolbachia presence affecting vesicular traffic, (IX) Wolbachia Rnase production, destroying viral RNA, (X) Wolbachia could also compete with arbovirus for resources like lipids inducing its storage as lipid droplets (LD), (XI) Wolbachia infection can change the interaction between the arbovirus and the insect specific viruses (ISV), (XII) potential effect of Wolbachia infection on the extracellular vesicles infection mechanism used by the arboviruses, (XIII) Changes of microbiota composition by the arbovirus infections, associated to the immunological response activation and AMPs production. **(B)** Interactions of previous viral infections with new arboviral infection, (I), immune priming mediated by interference RNA (iRNAs), (II) Endogenization of non-retroviral elements (NIRVS) from viral RNA and ISV RNA, through the insect autonomous transposable elements (TEs) and its retrotranscriptase (RT), creating viral intermediaries DNA (VDNA) for i RNA production, (III) Overexpression of proviral proteins for ISV infection, (IV) Synthesis of non-functional viral proteins, promoting non-functional viral particles, (V) subgenomic flaviviral RNAs (sfRNA) interfere with iRNAs responses, (VI) arbovirus infection can also change ISV presence (mostly enhance), probably by the changes on mosquito immunological response like interfering with iRNA response.

The viral envelope (E) protein binds to various mosquito cell receptors such as HSC70, laminin union proteins, enolase, and prohibitins, depending on the virus and cell type (Sakoonwatanyoo et al., 2006; Cheng et al., 2010; Kuadkitkan et al., 2010; Liu et al., 2014; Ghosh et al., 2018). Wide tissue expressing C-type lectins may also help the DENV E protein bind to mosquito tyrosine phosphatase receptors (Cheng et al., 2010). After binding, the virus is internalized via clathrin-mediated endocytosis (Figure 1B-2), and the drop in endosome pH triggers E dimer dissociation, exposing the hydrophobic domains and leading to membrane fusion and viral genomic RNA (vgRNA)-capsid release (Figure 1B-3). The A226V mutation in the E1 protein of CHIKV boosts infection of *A. albopictus* cells by increasing cholesterol affinity (Tsetsarkin et al., 2007).

The viral genomic RNA (vgRNA) of DENV, ZIKV, YFV, and CHIKV are positive-stranded and encodes a viral polyprotein, including non-structural and structural proteins. Non-structural proteins with protease activity release themselves, and other non-structural proteins, such as RNA-dependent RNA polymerase, are responsible for synthesizing vgRNA and subgenomic RNA (sgRNA). Post-translational modifications of E-related proteins occur in the endoplasmic reticulum, Golgi complex, and plasma membrane. In the cytoplasm, capsid proteins interact with the vgRNA to package it, and this complex moves to the cell membrane in the presence of E proteins to assemble and release viral particles (Velandia and Castellanos, 2011; Calvo et al., 2021).

Cell-secreted extracellular vesicles (EVs) have been proposed as a complementary mechanism through which arboviruses infect arthropods and vertebrates (Reyes-Ruiz et al., 2020; Sultana and Neelakanta, 2020). EVs are used for cell-to-cell communication and carry DNA, RNA, and proteins (Teng and Fussenegger, 2021), and EVs produced by infected mosquito cells have been reported to carry viral RNA fragments and proteins that can infect mosquitoes or mammalian cells. This infection strategy helps to reduce the cytopathic effect and avoid the immune response in mosquitoes and vertebrate hosts (Reyes-Ruiz et al., 2020). Additionally, the protein cargo of EVs changes in DENV-infected cells, increasing the presence of proteins that can enhance subsequent viral infections (Gold et al., 2020). These vesicles can be transported through cell-cell junctions (Cheng et al., 2020) or released into the extracellular space (Vora et al., 2018).

### 2.3 The mosquito immune response

The cellular response to viral pathogens involves phagocytosis and encapsulation of pathogens by hemocytes and the production of antimicrobial peptides (AMPs), reactive oxygen species (ROS), complement proteins (CP) such as opsonins, and thioester-containing proteins (TEPs) (Figure 1A). These responses are mediated through the Toll pathway (Figure 1A-I), the immune deficiency (Imd) pathway (Figure 1A-II), and the JAK/STAT signaling pathway (Figure 1A-III); their roles in the mosquito immune response have been well-described and reviewed previously (Shaul and Seger, 2007; Xi et al., 2008; Fragkoudis et al., 2009; Xiao et al., 2014; Cheng et al., 2016; Simões et al., 2018; Wang et al., 2019; Chowdhury et al., 2020; Liu et al., 2020; Rosendo Machado et al., 2021; Prince et al., 2023). It has been reported that these pathways are activated during DENV and ZIKV infections, but not CHIKV infections, in *A. aegypti* and *A. albopictus* cell lines (Xi et al., 2008; Houé et al., 2019a; Abduljalil and Abd Al Galil, 2022). Other additional pathways were identified in the *Aedes* mosquito cells. The first pathway is the mitogen-activated protein kinase (MAPK) pathway (Figure 1A-IV), mediated by receptor tyrosine kinase (RTK), which is activated by ROS (Horton et al., 2011; Plotnikov et al., 2011; Arthur and Ley, 2013), and signals Ras proteins to hydrolyze GTP, leading to Raf, MEK, and ERK activation and AMPs production (Shaul and Seger, 2007; Liu et al., 2020). The delta-Notch pathway (Figure 1A-V) activates Notch receptors in midgut cells, triggering Notch cleavage and endoreplication of genetic material without cell division, thereby increasing antiviral and immune gene expression (Serrato-Salas et al., 2018).

### 2.4 The interference RNA response

The main antiviral response in insects is mediated by interfering RNA (iRNA) such as small interfering RNA (siRNAs) (Figure 1B-I), PIWI-interacting RNA (piRNAs) (Figure 1B-II), and microRNAs (miRNAs) (Figure 1B-III) (Olson and Blair, 2015; Cheng et al., 2016; Tassetto et al., 2017; Silverman et al., 2019; Rosendo Machado et al., 2021; Dong et al., 2022). These responses are activated by the double-stranded RNA present in the secondary structures of vgRNA, RNA intermediates, or mosquito cell transcripts from specific genomic regions. They can be transferred to other cells via GAP junctions, cytoplasmic bridges, or EVs. EVs produced by Drosophila hemocytes release siRNA during localized tissue infection as an immunological response (Tassetto et al., 2017). Dicer2 expression during siRNA synthesis induces vago transcription (Kingsolver and Hardy, 2012).

Additionally, reverse transcriptase (RT) from autonomous transposable elements (TEs) can reverse transcribe viral RNA (vRNA) into Intermediate Viral DNA (vDNA). Dicer2 and its helicase subunit recognize and facilitate interactions between vRNA and RT. The vDNA is endogenized by the integrase produced by the same autonomous TEs to be integrated into non-retroviral endogenous elements (NERVE) (Figure 1B-IV) for piRNA and miRNA production or remains in the cytoplasmic region as linear or circular vDNA serving as a template for siRNA (Houé et al., 2019a). This adaptive immune mechanism can be inherited (Goic et al., 2016; Mukherjee et al., 2019; Mondotte et al., 2020). The presence of TEs is typically considered deleterious. However, TEs represent almost half of *A. aegypti* and *A. albopic*tus genomes (Houé et al., 2019b). Furthermore, TEs, especially autonomous TE-like long terminal repeats, appear to be active, as evidenced by the genetic richness of these elements in both mosquito species (de Melo and Wallau, 2020). This evidence suggests that despite the potential cost of TE activity, mosquitoes use it to develop adaptive immunity to tolerate arboviral infections.

### 2.5 The mosquito microbiota

Mosquitoes are holometabolous insects with differential exposure to microorganisms throughout their lives (Coon et al., 2014, 2016; Correa et al., 2018; Scolari et al., 2019; Tawidian et al., 2021), and their microbiota include bacteria, viruses, fungi, and archaea (Dickson et al., 2017, 2018; Öhlund et al., 2019; Tawidian et al., 2021). *A. aegypti* and *A. albopictus* breeding sites are usually small natural or artificial water bodies that can be clean or polluted (Chandrasiri et al., 2020). The physicochemical characteristics of the breeding site influence the richness and diversity of the microbial composition in the water and mosquitoes (Gusmão et al., 2007; Yadav et al., 2015; Bozic et al., 2017; Dickson et al., 2017; Mancini et al., 2018; Bogale et al., 2020; Tawidian et al., 2021).

The microbiota composition varies depending on the sex of the mosquito, developmental stage, larvae diet, blood source, ecology, geographical location, temperature, mosquito genetics, and metabolism (Minard et al., 2018; Parry et al., 2018; Bogale et al., 2020; Onyango et al., 2020; MacLeod et al., 2021; Pérez-Ramos et al., 2022; Sarma et al., 2022; Ratnayake et al., 2023; Rodpai et al., 2023). Water microbiota composition is also influenced by larval feces and the egestion of microorganisms by females during oviposition (Guégan et al., 2018; Scolari et al., 2019, 2021). However, most mosquito larval microbiota are lost in adults owing to meconium ingestion. Maternally transmitted microorganisms create a core microbiota associated with each species regardless of their geographic distribution (Yadav et al., 2015; Mancini et al., 2018; Scolari et al., 2021; Olmo et al., 2023). Wolbachia bacteria, *Microsporium* fungi, insect-specific viruses, and arboviruses are vertically transmitted microorganisms (Werren, 1997; Duguma et al., 2015; Velandia-Romero et al., 2017; Dickson et al., 2018; Scolari et al., 2019; Sicard et al., 2019).

Bacterial composition in adults are an important part of midgut microbiota with specific and common bacteria genera composition (Supplementary Figure 1) (Yadav et al., 2015; Raharimalala et al., 2016; Dickson et al., 2018; Hegde et al., 2018; Rosso et al., 2018; Thongsripong et al., 2018; Bennett et al., 2019; Arévalo-Cortés et al., 2020; Molina-Henao et al., 2020; Ramos-Nino et al., 2020; Seabourn et al., 2020; Balaji et al., 2021; Díaz et al., 2021; Lin et al., 2021; Scolari et al., 2021; Rau et al., 2022; Sarma et al., 2022; Al-Ghamdi et al., 2023; Baltar et al., 2023; Martinez Villegas et al., 2023; Mosso González et al., 2023; Rodpai et al., 2023). Wolbachia is the most characterized bacteria in *Aedes* mosquitos (Supplementary Table 1 and Supplementary Figure 1) (Kitrayapong et al., 2002; Ravikumar et al., 2010; Tortosa et al., 2010; de Albuquerque et al., 2011; Wiwatanaratanabutr, 2013; Joanne et al., 2015; Noor Afizah et al., 2015; Raharimalala et al., 2016; Ahmad et al., 2017; Nugapola et al., 2017; Soni et al., 2017; Chuchuy et al., 2018; Goindin et al., 2018; Hegde et al., 2018; Thongsripong et al., 2018; Anderson et al., 2019; Balaji et al., 2019; Carvajal et al., 2019; Kulkarni et al., 2019; Mohanty et al., 2019; Shaikevich et al., 2019; Ding et al., 2020; Hu et al., 2020; Puerta-Guardo et al., 2020; Torres-Monzón et al., 2020; Lin et al., 2021; Duque-Granda et al., 2022; Roslan et al., 2022; Sasaki et al., 2022; Zhang et al., 2022; Bueno-Marí et al., 2023; Chao and Shih, 2023; Li et al., 2023; Ruiz et al., 2023; Somia et al., 2023; Vinayagam et al., 2023). Due to its ability to affects insect reproduction and genetic diversity (Sicard et al., 2019; Sinha et al., 2019).

*A. albopictus* could have a *Wolbachia pipientis* prevalence of up to 90% in some regions, and wAlbA and wAlbB are the two prominent strains. Although *A*. aegypti it has been artificially infected with *Drosophila melanogaster* (wMel and wMelpop) and *A. albopictus* Wolbachia strains (Moreira et al., 2009; Walker et al., 2011), with reports of some populations with natural infections (Supplementary Figure 1) (Thongsripong et al., 2018; Balaji et al., 2019; Carvajal et al., 2019; Kulkarni et al., 2019; Zhang et al., 2022; Chao and Shih, 2023; Somia et al., 2023; Vinayagam et al., 2023).

Furthermore, mosquitoes are exposed to several arboviruses and insect-specific viruses (ISVs) (Supplementary Figure 2 and Supplementary Table 1). ISVs have been described in the Birnaviridae, Bunyaviridae, Mesoniviridae, Negeviridae, Nodaviridae, Reoviridae, Rhabdoviridae, Togaviridae, Tymoviridae, and Flaviviridae families. They only infect insects and are vertically transmitted (Bolling et al., 2015; Hoyos-López et al., 2016; Guzman et al., 2018; Salim Mattar and Marco González, 2018; Öhlund et al., 2019; Laiton-Donato et al., 2023).

Flaviviridae has the highest ISV richness, and it has been proposed that the arbovirus originates in ISV adapting to vertebrate hosts with the development of hematophagy by some arthropods (Bolling et al., 2015; Guzman et al., 2018; Öhlund et al., 2019). The specificity of ISV to invertebrate hosts is related to genetic, structural, immunological, and microclimatic conditions (Elrefaey et al., 2020). In addition, multiple infections with arboviruses such as DENV, ZIKV, YFV, and CHIKV have been found in field-collected mosquitoes, with evidence of vertical transmission (Velandia-Romero et al., 2017; Alencar et al., 2021; Mantilla-Granados et al., 2022). Thus, arboviruses are a part of the normal microbiota of these insect vectors, making them reservoirs.

### 2.6 Arbovirus-mosquito microbiota relationships

The midgut lumen is the mosquito compartment with the highest presence of microbiota, consisting mainly of commensal microorganisms that have developed different strategies to resist chemical and enzymatic activity, and the mosquito immune response mediated by broad-spectrum AMPs expressed to different pathogen-associated molecular patterns (PAMPS) (Caragata et al., 2019), which could favor or limit the development of arboviruses. For example, *Talaromyces* sp. produces specific metabolites that reduce the transcription of trypsin peptidases, restricting trypsin activity in the gut to ensure their development (Figure 2A-I). This indirectly favors DENV mosquito infections (Angleró-Rodríguez et al., 2017). Other gut mosquitoes fungus like *Zancudomyces culisetae* also change bacteria composition at midgut (Frankel-Bricker, 2020), probably affecting bacterial effect on arbovirus infection.

Mosquito commensal homeostatic bacteria can change after blood digestion, altering the bacterial diversity, fungus susceptibility, ROS, and AMPs production in mosquitoes (Xiao et al., 2017; Wang et al., 2019; Cabral et al., 2020). To protect themselves and restore gut microbiota homeostasis, bacteria alter lectin production through the Imd pathway, involving classical NF-κB immune signaling, to increase lectins, which protect the commensal microbiota from AMPs activity (Figure 2A-II) (Pang et al., 2016). Changes in lectin composition also affect the ability of arboviruses to infect mosquito cells. Similarly, *Serratia odorifera* infection increased the production of the P40 protein (Figure 2A-III), which mediates the interaction of CHIKV and DENV envelope proteins with cellular prohibitin receptors, favoring viral entry. Presence of ZIKV in blood-meal favors bacteria diversity, favoring some bacteria genera, probably through mosquito-immune regulation and changes on bacteriophage load (Shi et al., 2022).

*Serratia marcescens* gut bacteria secrete SmEnhancin (Figure 2A-IV), which weakens the mucin layer and increases DENV infectivity in *A. aegypti* (Wu et al., 2019), whereas *Chromobacterium* spp. aminopeptidases and lipase production (Figure 2A-V) degrade the DENV envelope (Ramirez et al., 2014; Yu et al., 2022), *S. marcescens* can also affects mosquito larval survival and adult microbiota composition (Heu et al., 2021). Midgut microorganisms limit arboviral development by activating immunological pathways and producing broad-spectrum AMPs, ROS, and opsonins (Xi et al., 2008). For instance, *Proteus* spp. increase AMPs production by activating the Toll pathway, thus reducing DENV infection (Figure 2A-VI), and *Beauveria bassiana* fungus in *A. aegypti* activates the JAK-STAT and Toll pathways, thereby increasing resistance to DENV (Dong et al., 2012). In contrast, DENV can induce AMPs production to reduce gut microbiota (Ramirez et al., 2012), ZIKV has also been reported to modulate microbiota composition (Arévalo-Cortés et al., 2022). Microbiota bacteria also affect the development of *Serratia* (Kozlova et al., 2021).

The effect of Wolbachia has different mechanism for restrict arbovirus transmission, indirect and mediated by innate immunological priming (Figure 2A-VII) (Pan et al., 2012). Additionally, Wolbachia hijacks the cellular resources from the host cells (e.g., amino acids, nucleotides, cell machinery, phospholipids, and cholesterol), affecting their availability (Frentiu, 2017; Geoghegan et al., 2017; Lindsey et al., 2018; Teramoto et al., 2019), interfering arbovirus infection, replication and assembly. Furthermore, Wolbachia causes endoplasmic reticulum stress, disrupting viral envelope protein processing. The bacteria also alters microtubule organization (Figure 2A-VIII) and vesicular trafficking (Ferree et al., 2005), a key factor in arbovirus entry (Velandia and Castellanos, 2011; Calvo et al., 2021), and Wolbachia wRNase HI degrades DENV RNA, suppressing early infection (Hussain et al., 2023b), Wolbachia could also affects EVs biogenesis and their involvement in arbovirus infection.

Not all Wolbachia strains have the same arboviral restriction. For example, wAlbA does not have an inhibitory effect, whereas wAlbB restricts salivary glands infection in *A. albopictus* (Mousson et al., 2012). Compared to wMel and wMelPop, wAlbB had a lower suppressive effect on DENV infection in *A. aegypti* (Flores et al., 2020). Additionally, Wolbachia changes midgut bacterial composition (Audsley et al., 2018), but increase ISV-flavivirus presence (Amuzu et al., 2018; Balaji et al., 2021). The wMel infection seems to be genetically stable in *A. aegypti*, as well as their levels of infection and their capacity to restrict arbovirus infection at population level (Frentiu et al., 2014; Ford et al., 2019; Ahmad et al., 2021). However, as the Wolbachia tissue infection is not homogeneous, its arbovirus restriction is cell-dependent, and the arbovirus restriction capacity in the mosquito is not complete and change among arbovirus and serotypes (Carrington et al., 2017; Flores et al., 2020; Fraser et al., 2020), this opens the possibility for emerging and selection of Wolbachia-resistant arboviruses. However, the likelihood of this event is low but not impossible (Edenborough et al., 2021), as Wolbachia use different mechanism to interfere with arbovirus development, it makes difficult for the virus to overcome all. At population level probably if Wolbachia-resistant arbovirus emerge, the performance of Wolbachia strategy to reduce arbovirus transmission will be compromise.

*A. aegypti* and *A. albopictus* are commonly infected with ISVs (Supplementary Figure 2 and Supplementary Table 1) (Hoshino et al., 2009; Rizzo et al., 2014; Vasilakis et al., 2014; Chandler et al., 2015; Fan et al., 2016; Fernandes et al., 2016; Sadeghi et al., 2017; Ajamma et al., 2018; Fang et al., 2018, 2021a,b; Iwashita et al., 2018; Parry et al., 2018, 2021; Zakrzewski et al., 2018; Zhang et al., 2018; Gravina et al., 2019; Martin et al., 2019, 2020; Shi et al., 2019; Baidaliuk et al., 2020; da Silva Ferreira et al., 2020; Diagne et al., 2020; Jeffries et al., 2020; Kubacki et al., 2020; Ramos-Nino et al., 2020; Ribeiro et al., 2020; Supriyono et al., 2020; Thannesberger et al., 2020; Batson et al., 2021; Chiuya et al., 2021; Hameed et al., 2021; He et al., 2021; Munivenkatappa et al., 2021; Nebbak et al., 2021; Yezli et al., 2021; Calle-Tobón et al., 2022; Coatsworth et al., 2022; Duarte et al., 2022; Oguzie et al., 2022; Palatini et al., 2022; Aragão et al., 2023; Bennouna et al., 2023) that may alter their vectorial competence (Bolling et al., 2015; Öhlund et al., 2019). Phasi Charoen-like virus (PCLV) and Humaita Tubiacanga virus (HTV) are common ISVs found in *A. aegypti*, and *A. albopictus* (Supplementary Figure 2). Furthermore, their cocirculation is linked to DENV endemic zones, and *in vitro* PCLV and HTV infection in mosquito cells increases the expression of histone 4, a proviral factor (Olmo et al., 2023). However, another study found that PCLV infection of Aag2 cells conferred a DENV infection-resistant phenotype after wAlbB was cleared, highlighting a complex relationship between ISV and Wolbachia (Hussain et al., 2023a). Mosquito densoviruses also decrease DENV2 vectorial competence in *A. albopictus* mosquitoes (Kong et al., 2023).

Previous exposure to inactivated DENV during the larval stage has been shown to confer protection against subsequent DENV infections in *A. aegypti* females, and their generations can inherit resistance (Mondotte et al., 2020; Vargas et al., 2020). The mechanism of this adaptive immunity appears to be mediated by siRNA, piRNA, and miRNA (Figure 2B-II) via viral DNA (vDNA) intermediates produced by RT from TE (Figure 1B-IV, Figure 2B-III) (Houé et al., 2019a). vDNA endogenization can also occur in ISV RNAs, some of which are genetically related to arboviruses and serve as templates for iRNAs against conserved arboviral RNA sequences for degradation. Another mechanism by which vDNA endogenization affects viral development is the production of defective viral proteins from endogenous vDNA (Houé et al., 2019a), which compete with normal viral proteins during viral assembly (Figure 2B-IV).

Arbovirus co-infections, even from different arbovirus families, such as Flaviviridae (DENV, ZIKV, and YFV) and Togaviridae (CHIKV), are possible in wild *Aedes* spp. mosquitoes (Mantilla-Granados et al., 2022). Arboviruses can exhibit different strategies to evade the immunological response of mosquitoes. Flaviviruses can limits the iRNA-mediated through subgenomic flaviviral RNAs (sfRNAs; Yeh and Pompon, 2018). These non-coding regions have complex secondary structures that interfere with helicases from the host cell, recruiting and kidnapping the machinery for the recognition and degradation of vRNA, thus helping the virus spread (Pompon et al., 2017; Yeh and Pompon, 2018; Göertz et al., 2019). This evasion mechanism is conserved in ISV flaviviruses (Slonchak and Khromykh, 2018; Slonchak et al., 2022). It is possible that co-infection with ISV could also facilitate subsequent arbovirus infections by reducing the activity of iRNA with their sfRNA, this could also help to evade response through vDNA. On the other hand experimental co-infections with two arbovirus (DENV2 and ZIKV) have shown mutual enhancement, mediated by the non-structural protein NS5 (Lin et al., 2023).

## 3 Perspectives

### 3.1 *In vitro* and laboratory models

To get a better understanding of the factors involving on vectorial competence in order to design better control strategies, it is important to clarify the role of EVs in arbovirus infections, immunological response and arbovirus transmission to the vertebrate hosts, using a comprehensive evaluation and *in vitro* experimentation (Théry et al., 2018). Studies on mosquito cell lines, as well as primary cultures of midgut epithelial cells, salivary glands, and circulatory cells, can help us understand EVs function in arbovirus transmission to mosquitoes of mammal cells, as well as in the immune response as was demonstrate for Drosophila (Tassetto et al., 2017). In addition, it is important to characterize the potential role of Wolbachia and other microorganism on EVs biogenesis and trafficking, as this could be other mechanism to interfere arbovirus transmission. Also, a deeply characterization of the mosquito adaptive immune response involving endogenous TEs and DNA endoreplication, are key pieces to understand the vectorial competence. Another point to take into account for *in-vitro* models is the presence of ISV in insect cell lines or primary cell cultures (Fujita et al., 2018). Finally, for *in-vivo* experiments, the use of gnotobiotic or axenic mosquitoes is a powerful strategy to understand the effect of single and multiple microorganisms in the whole mosquito and it's the vectorial competence, helping to validate *in vitro* and fieldwork results (Romoli et al., 2021; Wu et al., 2023).

### 3.2 Field characterization

The characterization of mosquitoes microbiota in terms of fungi, bacteria, and viruses from different regions is important (Supplementary Figures 1–3), to get a better knowledge of the common microbiota presence, geographical patterns and to detected associations with arbovirus transmission (Olmo et al., 2023), being important to use different molecular markers (Frankel-Bricker and Frankel, 2022; Schrieke et al., 2022), with special focus on fungus since its characterization remains scarce (Angleró-Rodríguez et al., 2017; Zakrzewski et al., 2018; Luis et al., 2019; Guégan et al., 2020; Ramos-Nino et al., 2020) and go further characterizing the transcriptomic, proteomic and metabolomic networks, that are driving the interactions among the microbiota, with the mosquito and arboviruses. Another important topic is how the mosquitoes exposure to antibiotics could change their microbioma affecting its vectorial competence (Garrigós et al., 2023).

### 3.3 Microbiota as biocontrol strategies

The use of Wolbachia (wMel) is a successful and promising strategy for reducing arbovirus transmission (Aliota et al., 2016; Flores and O'Neill, 2018; Velez et al., 2020). However, it is important to characterize the interference mechanism with arbovirus development, as well as the fitness costs of the bacteria in their new host, interaction with the mosquito microbiota at the local level, the effect of local climatic conditions on Wolbachia infection, and interaction with other components of the mosquito microbiota. As *A. albopictus* is naturally infected, a meticulous characterization of Wolbachia strains in global populations is required. Furthermore, it is extremely important to characterize the potential mechanisms of the arbovirus to overcome the microbiota restriction pathways, since information is still scarce, not only for Wolbachia but also for other bacteria, ISV, and fungi, to guarantee the long-term success of microbiota use as transmission control strategies. Another microorganism like the ISV PCLV and densovirus, as well as the fungus *Beauveria bassiana*, or Serratia bacteria, have the potential to be used as control strategies to reduce arbovirus transmission, but more studies have to be conducted to prove their feasibility. The ISVs are also a promising biocontrol strategy for developing mosquito-mediated vaccines, not only for humans but also for wildlife or for changing vectorial competence. For example, by modifying the flaviviral ISV Chaoyang virus and replacing its premembrane and envelope proteins with those of ZIKV, a mosquito-delivered vaccine was developed to induce protective immunity against ZIKV in female mice (Wen et al., 2022), also mosquito cell-derived CHIKV-like particles have shown potential for vaccine development (Tsai et al., 2023).

## Author contributions

JM-G: Conceptualization, Funding acquisition, Investigation, Methodology, Writing–original draft. JC: Conceptualization, Funding acquisition, Methodology, Supervision, Writing–review & editing. MV-R: Conceptualization, Funding acquisition, Methodology, Supervision, Writing–review & editing.

## Supplementary material

Supplementary Figure 1 Reported studies by august 2023, of bacterial microbiota in *Aedes aegypti* and *Aedes albopictus* adults, for database construction (Supplementary Table 1), only studies with well-established mosquitoes collection origin, adult characterization microbiota and bacteria genus level characterization were chosen. Maps were constructed using ArcGis Pro 2.8.3. The maps shows, the number studies reporting samples from each country. The most reported bacteria or the ones previously reported with arbovirus effect were chosen to be displayed at the map, for easily visual identification *Aedes aegypti* was marked green and *Aedes albopictus* pink.

Supplementary Figure 2 Reported studies by august 2023, of insect specific virus (ISV) microbiota in *Aedes aegypti* and *Aedes albopictus* adults, for database construction (Supplementary Table 1), only studies with well-established mosquitoes collection origin, adult characterization microbiota. Maps were constructed using ArcGis Pro 2.8.3. The maps shows, the number studies reporting samples from each country. The most reported ISV or the ones previously reported with arbovirus effect were chosen to be displayed at the map, for easily visual identification *Aedes aegypti* was marked green and *Aedes albopictus* pink.

Supplementary Figure 3 Reported studies by august 2023, of fungi microbiota in *Aedes aegypti* and *Aedes albopictus* adults, for database construction (Supplementary Table 1), only studies with well-established mosquitoes collection origin, adult characterization microbiota and fungi genus level characterization were chosen. Maps were constructed using ArcGis Pro 2.8.3. The maps shows, the number studies reporting samples from each country. The most reported fungi or the ones previously reported with arbovirus effect were chosen to be displayed at the map, for easily visual identification *Aedes aegypti* was marked green and *Aedes albopictus* pink.
